# A 55-Year-Old Man with Stage IV Squamous Cell Carcinoma of the Right Groin after External Beam Radiation for Testicular Cancer

**DOI:** 10.1155/2014/346247

**Published:** 2014-06-15

**Authors:** Christine Ibilibor, Jeremy Wells, Sravan Kavuri, Kelvin A. Moses

**Affiliations:** ^1^Division of Urology, Department of Surgery, Georgia Regents University, Augusta, GA 30912, USA; ^2^Division of Hematology and Oncology, Department of Medicine, Georgia Regents University, Augusta, GA 30912, USA; ^3^Department of Pathology, Georgia Regents University, Augusta, GA 30912, USA; ^4^Department of Urologic Surgery, Vanderbilt University Medical Center, MCN, A-1302, Nashville, TN 37232, USA

## Abstract

Treating testicular cancer with adjuvant radiation has been associated with a number of second malignancies affecting the genitourinary tract and retroperitoneal structures; however, there have been few reported cases of cutaneous second malignancies. We report the case of a man who developed stage IV squamous cell carcinoma (SCC) of a condyloma after orchiectomy and adjuvant radiation for testicular cancer. We also review relevant literature available to date. A 55-year-old Caucasian man presented to the hospital with a large growth at the right groin which had grown into his right thigh preventing ambulation. His past medical history was significant for right testicular cancer of unknown pathology treated with orchiectomy and adjuvant radiation twenty years ago. Punch biopsy of the lesion revealed superficially invasive squamous cell carcinoma. He underwent excision of the growth with subsequent Cisplatin, radiation boost, and Paclitaxel regimens. Despite an aggressive treatment regimen and an initial good response, the patient's cancer progressed requiring palliative care. It is unclear whether or not therapeutic radiation in this case promoted the conversion of the patient's condyloma to a malignant lesion. Further studies are required at this time to clarify the clinical implications of these findings.

## 1. Introduction

Testicular cancer is the most common malignancy in males between the ages of 15 and 34, with seminoma being more responsive to therapy than nonseminoma [1, 2]. The mainstay of treatment for stages I and II seminoma is radical orchiectomy with or without postoperative radiation to the retroperitoneal and ipsilateral iliac lymph nodes. This paradigm has led to a disease-free survival rate of greater than 95% in stage I and 80–95% in stage II disease [3, 4]. Second malignancies after adjuvant radiation for testicular cancer have been well documented in the literature [3–7]. A population based study using surveillance, epidemiology, and end results data reported a 19% increased risk of developing a second malignancy after external beam radiation therapy for stage I seminoma within a 15-year period [1]. The most common types of second malignancies include transitional cell carcinoma of the bladder and ureters, non-Hodgkin's lymphoma, retroperitoneal sarcomas, pancreatic cancer, hematologic malignancies, and gastrointestinal malignancies involving the stomach, small intestine, colon, and rectum [1–8]. However, there are few reported cases of cutaneous second malignancies [9]. We report the case of a man who developed stage IV squamous cell carcinoma (SCC) after orchiectomy and external beam radiation for likely seminomatous testicular cancer.

## 2. Case Presentation

The patient is a 55-year-old Caucasian male with a past medical history of hypertension, congestive heart failure, and right testicular cancer treated with orchiectomy and adjuvant radiation of unknown dose and treatment field in 1993 for presumed seminoma. He presented to the emergency room in 2012 with a painful right groin mass that had been increasing in size and had recently caused impaired ambulation.

At the time of his treatment for testicular cancer, the patient noted that he had a small 1 cm condylomatous growth on his right groin. Fifteen years after he completed his radiation therapy, the patient noted the growth increasing in size. Three months prior to presenting to the hospital, the patient indicated that the growth was growing more rapidly, had begun to bleed, became painful and foul-smelling, and invaded his right thigh, making ambulation difficult.

**Figure 1:**
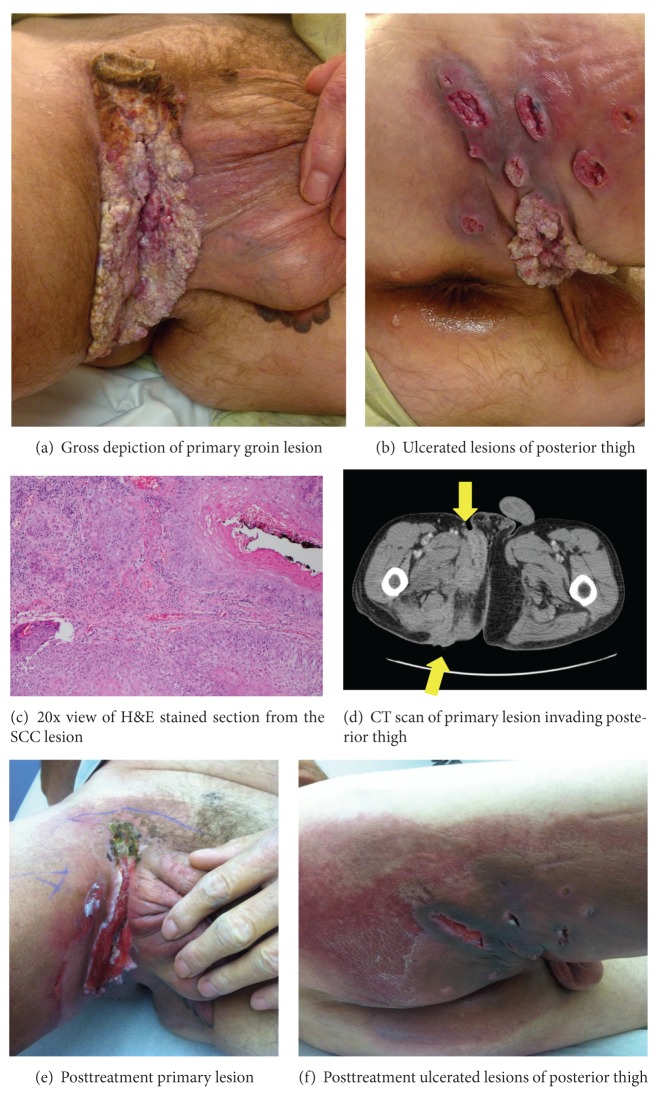
(a) Gross depiction of primary groin lesion. (b) Ulcerated lesions of posterior thigh. (c) 20x view of H&E stained section from the SCC lesion. (d) CT scan of primary lesion invading posterior thigh. (e) Posttreatment primary lesion. (f) Posttreatment ulcerated lesions of posterior thigh.

On physical exam, there was a foul-smelling, fungating growth which was located at the right inguinal ligament, extending across the groin into the perineum and posterior thigh (Figures 1(a) and 1(b)). The posterior aspect of the patient's upper thigh had six oval-shaped ulcerated lesions. Rectal examination did not reveal involvement of the rectal mucosa and the prostate was normal. His remaining exam was normal.

Laboratory testing revealed a hemoglobin of 10.7 g/dL and a hematocrit of 31.6%, and creatinine was 0.9. Punch biopsy of the lesion revealed superficially invasive SCC with verrucous features (Figure 1(c)). Further analysis found the lesion to be positive for low risk HPV; typing was not determined. An abdominal computerized tomography (CT) scan showed an enhancing soft tissue density beginning at the right groin extending through the perineum and into the posterior gluteal folds with involvement of the right quadriceps muscle (Figure 1(d)). The tumor depth was close to the femoral vessels as they cross the inguinal ligament. In Figure 1(d), the arrows indicate the infiltrating, invasive process involving the groin and posterior thigh. Positron emission tomography CT (PET-CT) showed uptake in the primary lesion and active lymphadenopathy in the inguinal regions bilaterally and left external iliac region. There was no evidence of distant metastases.

Weekly Cisplatin 20 mg/m^2^ for 6 weeks and weekly external beam radiation for a total dose of 4,500 Gy were initiated. After completion of this treatment, the lesion had reduced in size and exhibited changes consistent with tumor response (Figures 1(e) and 1(f)). PET-CT five months after presentation showed persistently hypermetabolic tumor involving skin and medial posterior thigh; thus, the patient received a radiation boost of 3,000 Gy. PET-CT ten months after presentation showed slightly more active primary tumor in the right inguinal and medial posterior thigh.

Considering the poor tumor response to the radiation boost, the patient was begun on weekly Cisplatin 70 mg/m^2^ for three weeks. A PET-CT twelve months after presentation showed progression of the malignancy. At this point, palliative care was offered to the patient; however, he chose continued care; thus, weekly Paclitaxel 80 mg/m^2^ for three weeks was initiated. After completion of the Paclitaxel regimen, there was minimal response and the patient accepted palliative care and was subsequently referred to hospice.

## 3. Discussion

There are reports of melanoma and nonmelanoma skin cancers developing outside and inside the radiation field in patients treated for seminomatous testicular cancer [6, 9]. It has been suggested that this may be due to the increased surveillance these patients are put under after treatment and previously undiagnosed lesions [7, 9]. To our knowledge, there are no reports of the development of cutaneous SCC in an irradiated and HPV infected lesion in a patient treated with radiotherapy for testicular cancer [5–7, 9].

Various risk factors have been implicated in the development of SCC including exposure to ultraviolet light, ionizing radiation, certain immunosuppressive therapies, and HPV infection [10–13]. There have been multiple studies regarding the risk of developing cutaneous SCC on areas previously exposed to therapeutic radiation in the treatment of acne, hemangiomas, and dermatitis [10].

Interestingly, some studies have shown that UV radiation exposure can work synergistically with HPV in the oncogenesis of SCC [13]. There has been one report of the development of cutaneous SCC of the nipple in a patient irradiated for breast cancer. In that report, the patient developed a nonhealing ulcer of the right nipple nine years after lumpectomy and completion of radiation for ductal carcinoma in situ; the ulcer was found to be invasive SCC. The authors of the case assert that the patient likely developed radiation dermatitis of the right nipple which predisposed it to developing invasive SCC; the patient had no previous lesions on the nipple before radiotherapy [12].

Although the radiation dose and field used to treat the initial testicular tumor of this patient are unknown, it is likely that he received radiation to the commonly included areas in stage I disease, namely, the para-aortic and ipsilateral iliac nodes (dog-leg or hockey-stick fashion) [3]. It is possible that the skin of the inguinal region fell within the targeted area for the ipsilateral iliac nodes. Considering the paucity of studies regarding the development of SCC within an irradiated field, it is unknown whether this patient's condylomatous lesion developed into a carcinoma by an independent process or was a radiation induced malignancy.

HPV infected skin lesions are quite ubiquitous; however, data indicating that therapeutic radiation causes transformation of these lesions into invasive SCC are lacking. More studies are needed to determine the role that therapeutic radiation exposure may have in the development of SCC in HPV infected lesions. Additionally, it is important to advocate the appropriate utilization of active surveillance for patients with clinical stage I seminoma to avoid the risks of secondary malignancy and overtreatment.

In conclusion, we present a case of invasive SCC after orchiectomy and radiotherapy for testicular cancer of unknown pathology. A multidisciplinary approach was utilized in order to gain tumor control and the patient had greater than one-year survival from time of diagnosis. However, disease progression occurred despite multiple chemotherapy and radiation regimens. It is unclear whether or not the transformation of this patient's original condyloma to invasive carcinoma was accelerated by radiation exposure or developed independently.
